# Enhanced photocatalytic reduction of CO_2_ to CO over BiOBr assisted by phenolic resin-based activated carbon spheres†

**DOI:** 10.1039/c9ra01329f

**Published:** 2019-05-08

**Authors:** Kangli Liu, Xiaochao Zhang, Changming Zhang, Guangmin Ren, Zhanfeng Zheng, Zhiping Lv, Caimei Fan

**Affiliations:** College of Chemistry and Chemical Engineering, Taiyuan University of Technology Taiyuan 030024 PR China zhangxiaochao@tyut.edu.cn +86-351-6018554 +86-155-03477962; State Key Laboratory of Coal Conversion, Institute of Coal Chemistry, Chinese Academy of Sciences China

## Abstract

Photocatalytic reduction of CO_2_ using solar energy to decrease CO_2_ emission is a promising clean renewable fuel production technology. Recently, Bi-based semiconductors with excellent photocatalytic activity and carbon-based carriers with large specific surface areas and strong CO_2_ adsorption capacity have attracted extensive attention. In this study, activated carbon spheres (ACSs) were obtained *via* carbonization and steam activation of phenolic resin-based carbon spheres at 850 °C synthesized by suspension polymerization. Then, the BiOBr/ACSs sample was successfully prepared *via* a simple impregnation method. The as-prepared samples were characterized by XRD, SEM, EDX, DRS, PL, EIS, XPS, BET, CO_2_ adsorption isotherm and CO_2_-TPD. The BiOBr and BiOBr/ACSs samples exhibited high CO selectivity for photocatalytic CO_2_ reduction, and BiOBr/ACSs achieved a rather higher photocatalytic activity (23.74 μmol g^−1^ h^−1^) than BiOBr (2.39 μmol g^−1^ h^−1^) under simulated sunlight irradiation. Moreover, the analysis of the obtained results indicates that in this photocatalyst system, due to their higher micropore surface area and larger micropore volume, ACSs provide enough physical adsorption sites for CO_2_ adsorption, and the intrinsic structure of ACSs can offer effective electron transfer ability for a fast and efficient separation of photo-induced electron–hole pairs. Finally, a possible enhanced photocatalytic mechanism of BiOBr/ACSs was investigated and proposed. Our findings should provide new and important research ideas for the construction of highly efficient photocatalyst systems for the reduction of CO_2_ to solar fuels and chemicals.

## Abstract 2

Photocatalytic reduction of CO_2_ using solar energy to decrease CO_2_ emission is a promising clean renewable fuel production technology.

## Graphical abstract

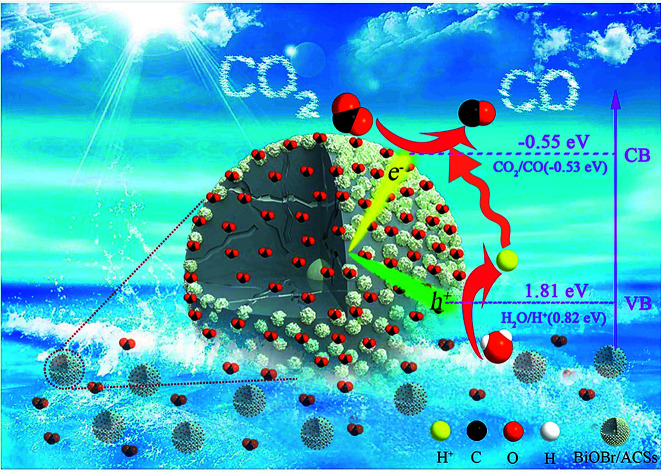

## 1. Introduction

With the rapid development of industrial economy, the global environmental pollution and energy crisis have become extremely significant challenges that humans are facing in the short-term.^1^ More importantly, the heavy reliance of economic development on the fossil fuel energy will bring about more than 30.4 Gt a^−1^ of CO_2_ emissions into the atmosphere,^2^ and the CO_2_ emissions will probably increase up to 43 Gt a^−1^ by 2035 without factitious CO_2_ conversion utilization. Moreover, CO_2_ has been considered as a dominating factor for the anthropogenic climate change to cause the “Greenhouse Effect”; therefore, it is necessary to find out suitable ways for the maximum utilization of CO_2_.^3,4^ At present, solar photocatalytic technology, as a great potential way to solve the abovementioned problem, has received significant attentions for the reduction of CO_2_ to useful high-energy fuels (*e.g.* CO, CH_3_OH, CH_4_, HCOOH, *etc.*).^5–10^ Among them, CO, as a crucial feedstock in the d-metal-catalyzed Fischer–Tropsch synthetic processes, possesses a significant fuel value (Δc*H* = −283.0 kJ mol^−1^) and is also easily converted to CH_3_OH; therefore, it is attracting significant interest from researchers.^11^ Most importantly, it is imperative to find a suitable and promising photocatalyst system with high selectivity and activity for CO_2_ reduction under the action of solar light.

BiOBr, as one of the Bi-based semiconductors with several advantages, including earth abundance, stability, economy and non-toxicity, exhibits a unique layered structure, excellent electrical and optical properties as well as a suitable indirect band gap (∼2.7 eV) to endow effective photocatalytic activity and stability.^12–17^ It was believed that the Bi 6s and O 2p states could form a large number of dispersed hybridized valence bands, which facilitated the migration and oxidation of photo-generated holes; this induced the efficient separation of photo-generated electron–hole (e^−^–h^+^) pairs and then improved the photocatalytic efficiency.^18^ In 2016, Ye *et al.* found that the as-prepared BiOBr sample with ultrathin thickness and a Bi-rich structure could convert CO_2_ to CO (2.67 μmol g^−1^ h^−1^) and CH_4_ (0.16 μmol g^−1^ h^−1^).^19^ After this, in 2017, they reported that {001}-dominated BiOBr nanosheets showed 100% selectivity for the conversion of CO_2_ to CO and the highest CO yield of 4.45 μmol g^−1^ h^−1^ under simulated sunlight irradiation.^20^ Most importantly, in 2018, Xie *et al.* synthesized defect-engineered BiOBr atomic layers with a rather high CO yield (87.4 μmol g^−1^ h^−1^) for the photocatalytic reduction of CO_2_ under visible light-driven irradiation.^21^ These remarkable findings well confirmed that BiOBr, as a promising new-type of CO_2_ reduction material, could be responsible for the excellent selectivity and activity of the photocatalytic reduction of CO_2_ to CO. As is known, the separation efficiency of photogenerated e^−^–h^+^ pairs and the adsorption performance of CO_2_ on the catalyst surface have been two significant and crucial factors for the photocatalytic reduction of CO_2_.^4,22–24^ However, for a single BiOBr photocatalyst, the low separation efficiency of the electron–hole pairs and the weak CO_2_ adsorption capacity limit its development and applications in the photocatalytic reduction of CO_2_. Therefore, it is urgent to construct an ideal photocatalyst system with excellent light absorption, efficient separation of photogenerated e^−^–h^+^ pairs and high CO_2_ adsorption capacity.^25^

According to abovementioned problems, the selection of materials with higher adsorption capacity, larger specific surface area and stronger charge transfer ability to be loaded onto high-efficiency photocatalysts should be a very significant and interesting issue in the research and development of photocatalytic CO_2_ reduction; in most of the studies, it has been found that carbon-based carriers (such as activated carbon,^26^ carbon nanotubes,^27^ graphene,^28^ and carbon nanodots^29^) meet these requirements due to their unique electronic structures, stable chemical structures, large specific surface areas, strong adsorption capacities, high thermal conductivity and electron mobilities.^30^ Especially, activated carbon spheres (ACSs) with excellent electron transfer capability, suitable sphere-type structure and size, high loading density and mechanical strength, have attracted widespread attention as a kind of promising and valuable adsorbent for hazardous materials in the liquid and gas phases as well as catalyst supports. For example, Liu *et al.* found that micrometer-sized carbon spheres, rich in O-containing functional groups, exhibited a remarkably enhanced adsorption capacity for Cr(vi), 0.4834 mmol g^−1^, about 4 times that of unmodified AC.^31^ Wickramaratne *et al.* prepared a series of activated carbon spheres (ACSs) through the carbonization of phenolic resin spheres using the one-pot modified Stöber and CO_2_ activation method, which exhibited high surface areas (from 730 to 2930 m^2^ g^−1^) and CO_2_ adsorption capacities at 1 bar (4.55 and 8.05 mmol g^−1^, at 25 °C and 0 °C, respectively).^32^ Moreover, Rivera-Utrilla *et al.* and Ao *et al.* synthesized TiO_2_-AC^33^ and BiOBr-AC^26^ photocatalysts, respectively, with greatly improved photocatalytic performances owing to the higher BET surface area of the AC support with strong adsorption capacity for pollutants and significant influence on the optical absorption capacity and crystal size of the catalysts. Therefore, due to their high specific surface areas and strong CO_2_ adsorption capacity, ACSs can become rather ideal carriers to support high-efficiency photocatalysts to effectively enhance the performance of photocatalytic CO_2_ reduction.

Among them, phenolic resins have been considered as promising polymer precursor materials to produce ACSs with high surface areas because of their low inorganic impurity and negligible ash contents. In our study, a new method is reported for the preparation of ACSs. At first, phenolic resin-based spheres were synthesized by suspension polymerization using *m*-cresol and formaldehyde as precursor materials and adding ethylene glycol or poly(ethylene glycol) as an additive; then, the millimeter-sized ACSs with high specific surface area were prepared by the steam activation method. Subsequently, a layer of BiOBr was uniformly loaded on the ACSs surface using a simple impregnation method. The phase structure, morphology, elemental analysis, optical absorption characteristics, specific surface areas and CO_2_ adsorption ability of the as-prepared BiOBr/ACSs were analyzed by XRD, SEM, EDX, XPS, DRS, PL, EIS, BET, CO_2_ adsorption isotherm and CO_2_-TPD. In addition, the correlative performances of photocatalytic CO_2_ reduction were evaluated under simulated sunlight irradiation. Finally, the photocatalytic mechanism of action of the BiOBr/ACSs sample has been investigated and proposed; the results obtained herein should provide new and important research ideas and great guiding significance for the construction of highly-efficient photocatalyst systems for photocatalytic CO_2_ reduction.

## 2. Experimental

### 2.1 Chemicals

Bismuth nitrate pentahydrate (Bi(NO_3_)_3_·5H_2_O) and potassium bromide (KBr) were purchased form Tianjin Sinopharm Chemical Reagent (Co. Ltd.) and Tianjin Zhiyuan Reagent (Co. Ltd.), respectively. *m*-Cresol was obtained from Aladdin. Formaldehyde, ethylene glycol (EG), and hexamethylenetetramine were provided by Tianjin Kemiou Chemical Reagent (Co. Ltd.). Triethylamine and polyvinyl alcohol were provided by Tianjin Fangde Technology (Co. Ltd.) and Shanxi Sanwei Group (Co. Ltd.). All reagents were of analytical grade. Deionized water was used throughout this study.

### 2.2 Preparation

#### BiOBr

In a typical synthesis, 0.01 mmol of Bi(NO_3_)_3_·5H_2_O was dissolved in 40 mL of ethylene glycol (EG) at 50 °C, and 0.01 mmol of KBr was dissolved in 20 mL of deionized water under continuous stirring until the solution became transparent. Subsequently, KBr was poured into the abovementioned solution. After stirring for 5 h, the suspension was filtered, repeatedly washed with deionized water and absolute ethanol, and finally dried at 60 °C overnight.

#### ACSs

A three-necked flask was set in an oil bath, and 200 mL of deionized water was added to it. Then, 30 mL of *m*-cresol, 33 mL of formaldehyde, 15 mL of ethylene glycol and 1.5 mL of trimethylamine were successively added to the three-necked flask. After this, 1.5 g of polyvinyl alcohol and 1.8 g of hexamethylenetetramine were added under continuous stirring when the temperature increased to 110 °C. After stirring for 3 h, the phenolic resin sphere samples were poured out, obtained, heated at 850 °C under a N_2_ atmosphere, and then activated by steam for 1 h to obtain ACSs.

#### BiOBr/ACSs

**Scheme 1:**
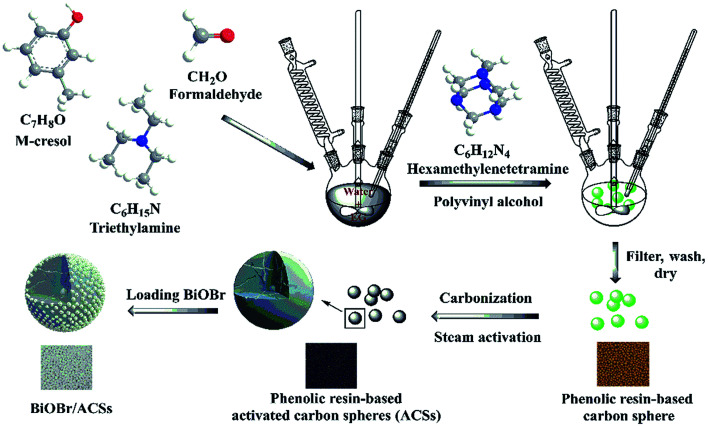
Preparation process of BiOBr/ACSs.

The BiOBr/ACSs samples were prepared by an impregnation method. Typically, 2 g of ACSs was added to a 0.05% polyvinyl alcohol solution and stirred for 1.5 h. The product was obtained by filtration. After this, 1 g of BiOBr was added to 20 mL of deionized water, the mixture was stirred for 10 min and ultrasonicated for 10 min, such that BiOBr was evenly dispersed in deionized water. The ACSs were added to the BiOBr solution and stirred for 20 min. After the reaction was completed, the product was filtered and dried at 60 °C for 12 h. The samples obtained were named BiOBr/ACSs, as shown in Scheme 1.

### 2.3 Characterization

The crystal phase of the as-prepared samples was investigated by X-ray diffraction (XRD) (DX-2700 diffractometer, Japan) with Cu Kα radiation in the 2*θ* range of 10–80°. The morphologies and element mapping of the prepared samples were investigated *via* the Nanosem 430 (FEI, America) field emission scanning electron microscope at the operating voltage of 10 kV with an energy dispersive X-ray (EDX) microanalysis system. XPS spectra of these materials were obtained *via* Thermo Fisher ESCALAB 250 xi, England, using Al Kα radiation (1486.6 eV). Binding energies were calculated with respect to C(1s) at 284.8 eV. Binding energies were measured with a precision of ±0.05 eV. The UV-vis diffuse reflection spectra (UV-vis DRS) were obtained using a UV-vis spectrophotometer (UV-3600, Shimadzu, Japan). Photoluminescence (PL) spectra were obtained using the Hitachi F-7000 Fluorescence spectrophotometer. The specific surface area and porous structure of all the samples were determined by N_2_ adsorption/desorption using the V-Sorb 4800P instrument. The CO_2_ adsorption isotherm was obtained using an automated gas sorption analyzer (Quantachrome Autosorb-1, USA). Temperature-programmed desorption of CO_2_ (CO_2_-TPD) was measured as follows: 0.1 g of sample was pretreated at 400 °C for 1 h under a He atmosphere and then naturally cooled down to 30 °C. Next, the samples were placed under a CO_2_ atmosphere for 0.5 h to ensure sufficient CO_2_ adsorption. Before desorption, the sample was purged with He for 3 h and then desorbed at the rate of 10 °C min^−1^ to 350 °C under a He atmosphere.

### 2.4 Photocatalytic CO_2_ reduction

Photocatalytic CO_2_ reduction experiments were carried out in a gas-closed circulation system (CEL-SPH2N-D9, Beijing China Education Au-Light Co., Ltd.) irradiated with a 300 W xenon lamp. Herein, 50 mL of deionized water was added to the quartz glass reactor, and a certain amount of catalyst was uniformly dispersed in the quartz glass reactor. Before turning on the light, the photoreactor system needed a thorough vacuum treatment, and CO_2_ gas was introduced into the circulation system. During the light irradiation of 9 h, the yield of CO produced was analyzed every hour by on-line gas chromatography (GC-7920, Beijing China Education Au-Light Co., Ltd.) with a flame ionization detector. Gas products were analyzed by an external standard method.

## 3. Results and discussion

### 3.1 Phase structure analysis

**Fig. 1:**
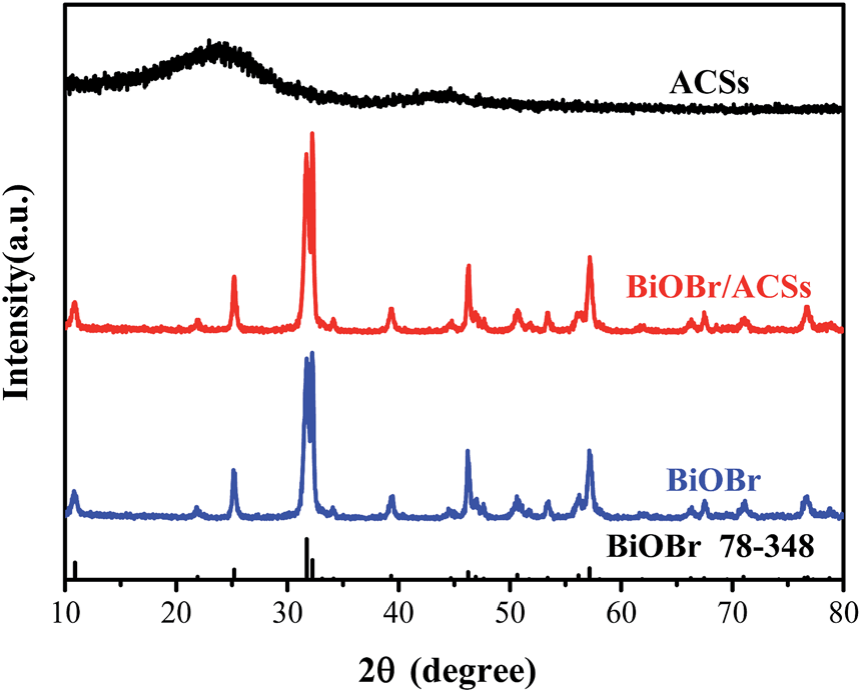
XRD patterns of ACSs, BiOBr/ACSs and BiOBr samples.

Fig. 1 shows the XRD patterns of the as-prepared BiOBr, ACSs, and BiOBr/ACSs samples. The XRD peaks of the BiOBr sample are well indexed to the tetragonal phase BiOBr (JCPDF no. 73-348),^13^ and no impurity peak is detected; this indicates high purity of as-prepared BiOBr sample. It has been found that the XRD patterns of BiOBr obtained after loading BiOBr onto ACSs do not shift significantly; this implies that the effect of ACSs on the phase structure of the BiOBr sample is negligible.

### 3.2 Morphological structure and energy dispersive X-ray (EDX) analysis

**Fig. 2:**
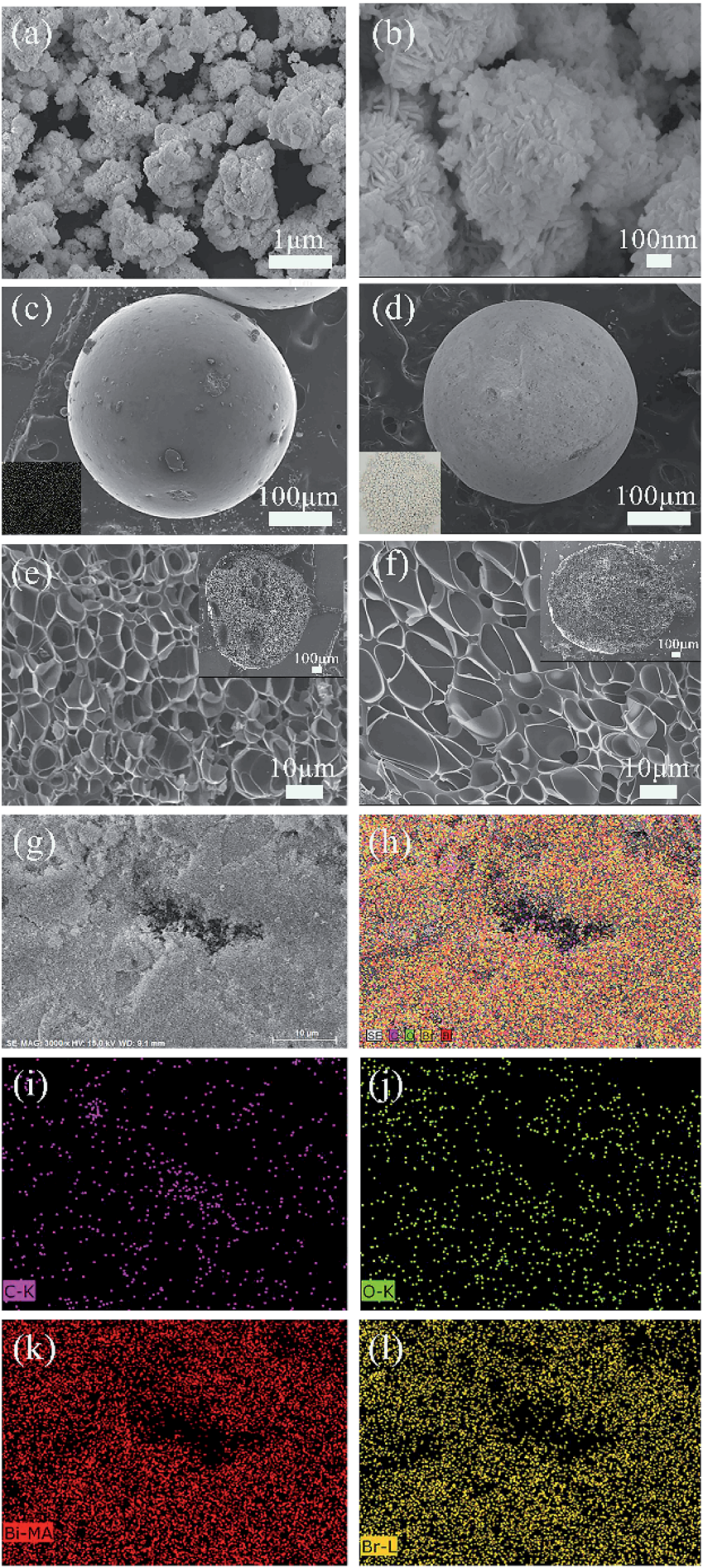
Low (a) and high (b) magnification SEM images of BiOBr, low-magnification SEM images of ACSs (c) and BiOBr/ACSs (d) (inset c: an image of ACSs; inset d: an image of BiOBr/ACSs), a high-magnification SEM image of ACSs (e) and BiOBr/ACSs (f) cross profiles (inset: low-magnification SEM images of ACSs (e) and BiOBr/ACSs (f) cross profile); (g and h) EDX mappings of the BiOBr/ACSs samples, C (i), O (j), Bi (k), and Br (l) elements.

Fig. 2 shows the SEM images of the prepared samples. In Fig. 2(a and b), it is found that the BiOBr sample exhibits a self-assembled sphere-structure with the single sphere diameter of approximately 0.8–1.2 μm. Moreover, the surface of the ACSs activated by steam is smooth and free from cracks with good sphericity (Fig. 2c), and an excellent pore-structure distribution is obtained *via* the carbonization process (inset in upper-right corner of Fig. 2e); the abovementioned conditions are favorable for the activation agent to enter the interior of the sample during the activation process, and finally, the formation of ACSs with different, new and expanding pore-distributions is achieved. For the BiOBr/ACSs sample (Fig. 2d), a large amount of white BiOBr powder obviously adheres to the ACSs surface (Fig. S1†), and BiOBr loading has almost no influence on the internal pore-structure distribution of ACSs, as shown in the partially magnified half-section of BiOBr/ACSs (Fig. 2f). In the high-magnification SEM image of BiOBr/ACSs (Fig. S1†), we can observe that BiOBr supported on the surface of ACSs still maintains its self-assembled spherical nanosheet structure; this indicates that there is almost no change in the morphology of BiOBr and ACSs before and after loading.

To further clarify the chemical compositions of the samples, the corresponding energy dispersive X-ray spectroscopy (EDX) elemental mappings (Fig. 2g–h) and EDX spectra (Fig. S2†) of the C, O, Bi and Br (Fig. 2i–l) elements were obtained. The atomic ratio of the Bi, O and Br elements is very close to the reasonable value of 1 : 1 : 1, and the observed Bi, O and Br elemental mappings well indicate that BiOBr can be evenly distributed and wrapped on the ACSs surface; thus, the excellent BiOBr/ACSs catalyst system can be formed.

### 3.3 XPS spectra

**Fig. 3:**
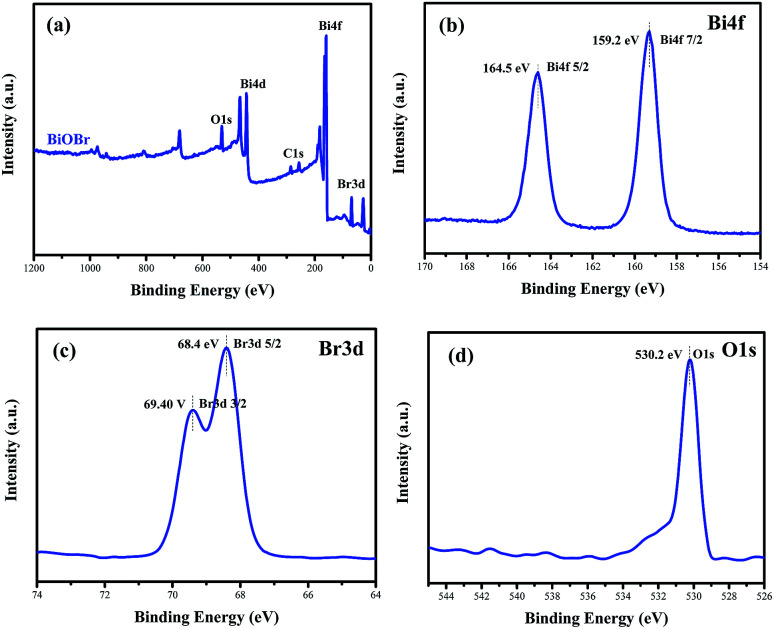
Survey XPS spectra (a) and high-resolution XPS spectra of BiOBr: Bi 4f (b), Br 3d (c) and O 1s (d).

To confirm the elemental compositions and valence states of the as-prepared samples, XPS survey spectra of the BiOBr sample were obtained, as shown in Fig. 3. It can be clearly seen from the wide survey scan data shown in Fig. 3a that Bi, O and Br are present without other prominent impurities; this rules out the possibility of the existence of adventitious carbon-based contaminants, and this is in good agreement with the abovementioned EDX scan results. The high-resolution XPS Bi 4f spectrum of BiOBr is presented in Fig. 3b. The two peaks at 164.5 and 159.2 eV are associated with Bi 4f_5/2_ and Bi 4f_7/2_, respectively, indicating that the Bi^3+^ valence state exists in the BiOBr nanosheets. As shown in Fig. 3d, the O 1s core level spectrum can be well fitted with the peak at 530.2 eV that belongs to the lattice oxygen O^2−^ originating from the Bi–O bond.^34^ The XPS spectrum of Br 3d (Fig. 3c) exhibits two major peaks with the binding energies at 68.4 and 69.4 eV, corresponding to Br 3d_5/2_ and Br 3d_3/2_ of Br^−^.

### 3.4 Optical absorption properties

**Fig. 4:**
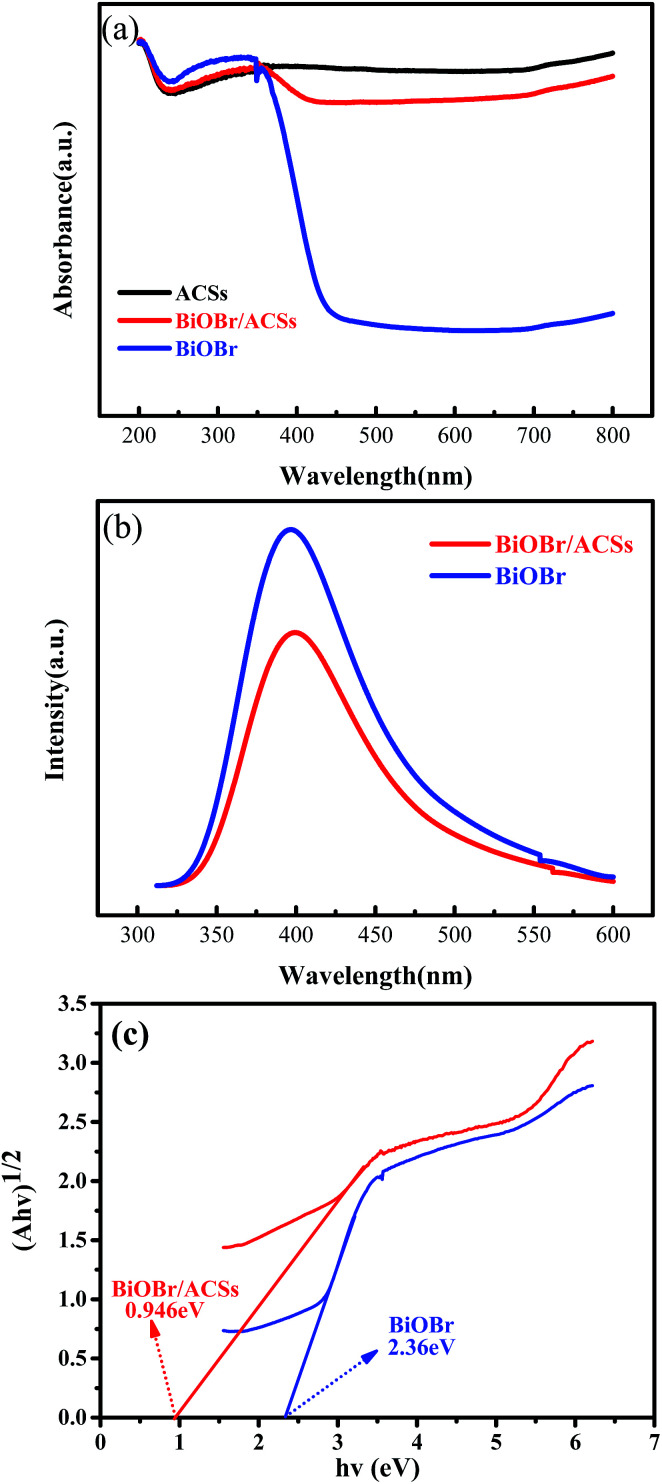
(a) DRS of the as-prepared samples; (b) PL spectra of BiOBr and BiOBr/ACSs samples; and (c) plots of (*αhν*)^1/2^*versus* photon energy (*hν*).

The optical absorption properties of the samples were analyzed *via* the DRS technology and PL spectroscopy, as illustrated in Fig. 4. The band gap (*E*_g_) of BiOBr and BiOBr/ACSs can be calculated by the equation (*αhν*)*n* = *A*(*hν* − *E*_g_).^35,36^Fig. 4c displays the curve of (*αhν*)^1/2^*vs. hν* according to the obtained BiOBr and BiOBr/ACSs DRS spectra, and the extrapolation intercept provides the *E*_g_ values for BiOBr and BiOBr/ACSs of 2.36 and 0.946 eV, respectively. After the introduction of ACSs, it can be clearly found that the BiOBr/ACSs sample has stronger optical absorption in the visible region than pure BiOBr. Thus, the addition of ACSs could improve the visible light-driven efficiency of BiOBr, enhancing the photocatalytic activity of BiOBr. Moreover, for the photocatalytic CO_2_ reduction process, the separation efficiency of photo-generated carriers should be an important factor. Herein, the PL spectra were obtained to evaluate the separation efficiency of the photo-generated e^−^–h^+^ pairs for the BiOBr and BiOBr/ACSs samples, as shown in Fig. 4b. As is known, the higher the PL intensity, the higher the probability of recombination of photo-generated e^−^–h^+^ pairs. It can be found that both pure BiOBr and BiOBr/ACSs samples have a diffraction peak at 400 nm; however, the latter reveals decreased intensity when compared with the former; this confirms an improved separation efficiency of the e^−^–h^+^ pairs after the introduction of ACSs.^37^ The EIS measurement results (Fig. S3†) reveal that the Nyquist circle of BiOBr/ACSs is smaller than that of BiOBr; this indicates that BiOBr/ACSs have lower resistance than pure BiOBr; this can accelerate the interfacial charge-transfer process.^38^

### 3.5 CO_2_ adsorption performance and porous structure parameters

**Fig. 5:**
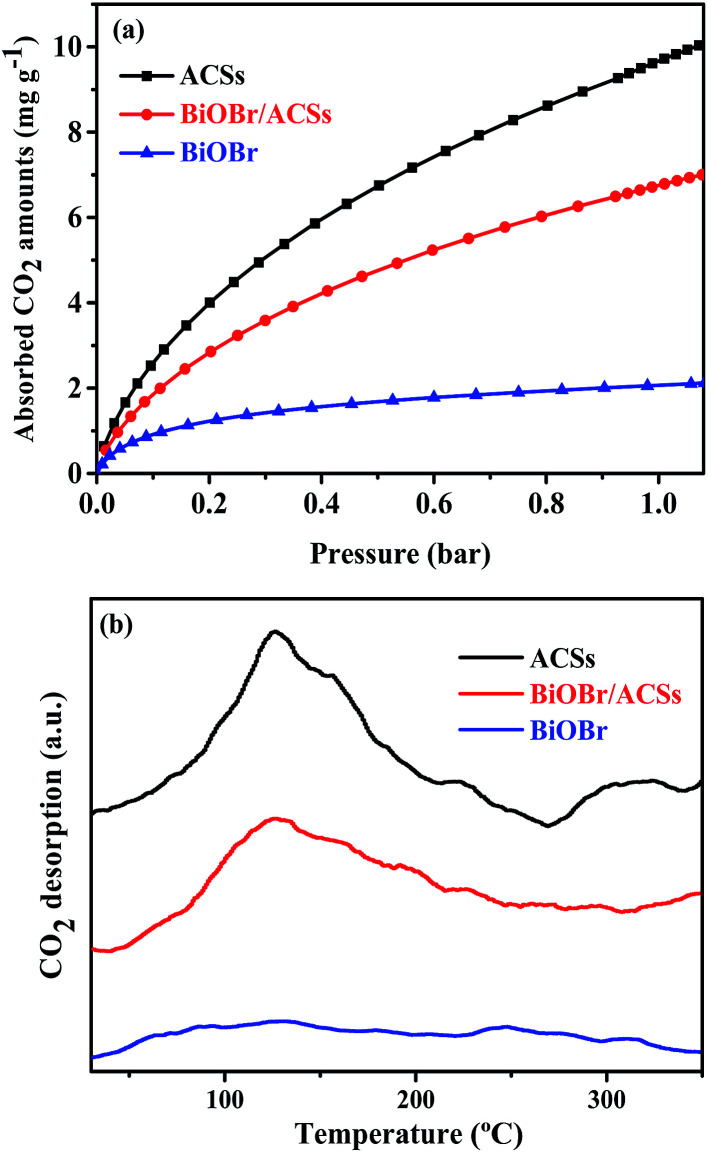
(a) CO_2_ adsorption isotherms and (b) CO_2_ temperature-programmed desorption (CO_2_-TPD) of the as-prepared samples.

The CO_2_ adsorption performance of a catalyst has been considered as one of the most key factors influencing the catalytic activity in the photocatalytic CO_2_ reduction process.^39^ The CO_2_ adsorption isotherms and CO_2_ temperature-programmed desorption (CO_2_-TPD) spectra for the BiOBr, ACSs and BiOBr/ACSs samples are provided in Fig. 5. The CO_2_ adsorption isotherms of the as-prepared sample at 298 K are shown in Fig. 5a. It is clearly seen that the CO_2_ adsorption capacity at 298 K over ACSs can reach 10.03 mg g^−1^, much higher than that of BiOBr/ACSs (7.0 mg g^−1^) and BiOBr (2.23 mg g^−1^); this should be ascribed to higher specific surface area and larger micropore volume of the ACSs and BiOBr/ACSs. In Fig. 5b, it can be found that pure ACSs possess strongest CO_2_ adsorption capacity, and the desorption peaks of CO_2_ occur at 50–150 °C and 260–330 °C, which should be mainly attributed to strong physical adsorption and weak chemisorption, respectively.^40,41^ In addition, pure BiOBr exhibits a notably weak CO_2_ desorption curve; however, after loading BiOBr on the ACSs surface, the intensity of the CO_2_ adsorption and desorption curves of the BiOBr/ACSs sample is obviously enhanced; this will be conducive to make an increasing number of CO_2_ molecules participate in the surface-activation and catalytic reaction process; this will improve the catalytic reaction efficiency and rate.^42^

Usually, a material with high surface area, large micropore volume and more basic surface functions should have higher CO_2_ capture performance.^43,44^Table 1 summarizes the corresponding structural parameters, including BET surface areas, average pore sizes, micropore and total pore volumes, as calculated from the N_2_ adsorption isotherms, of all the as-prepared samples. As can be observed from Table 1, the ACSs and BiOBr/ACSs samples have rather the high specific BET surface areas of 936.42 and 792.56 m^2^ g^−1^, total pore volumes of 0.37 and 0.31 cm^3^ g^−1^, and a high micropore ratio (>83%), respectively. These higher micropore surface area and larger micropore volume can provide more physical activation sites for CO_2_ adsorption, and the kinetic diameter of CO_2_ is 0.33 nm such that the CO_2_ adsorption capacity should ultimately be controlled by the micropore volume.^43,45^ These are the main reasons why ACSs exhibit higher CO_2_ adsorption ability than the BiOBr/ACSs and BiOBr samples.

**Table 1:** Porous structure parameters of the as-prepared BiOBr, ACSs and BiOBr/ACSs samples

Samples	*S*_BET_ (m^2^ g^−1^)	*V*_total_ (cm^3^ g^−1^)	*V*_micro_ (cm^3^ g^−1^)	*V*_semi_ − *V*_micro_ (cm^3^ g^−1^)	*D*_p_ (nm)
ACSs	936.42	0.37	0.34	0.32	1.58
BiOBr/ACSs	792.56	0.31	0.26	0.25	1.56
BiOBr	21.13	0.11	0.008	—	20.82

### 3.6 Photocatalytic CO_2_ reduction

**Fig. 6:**
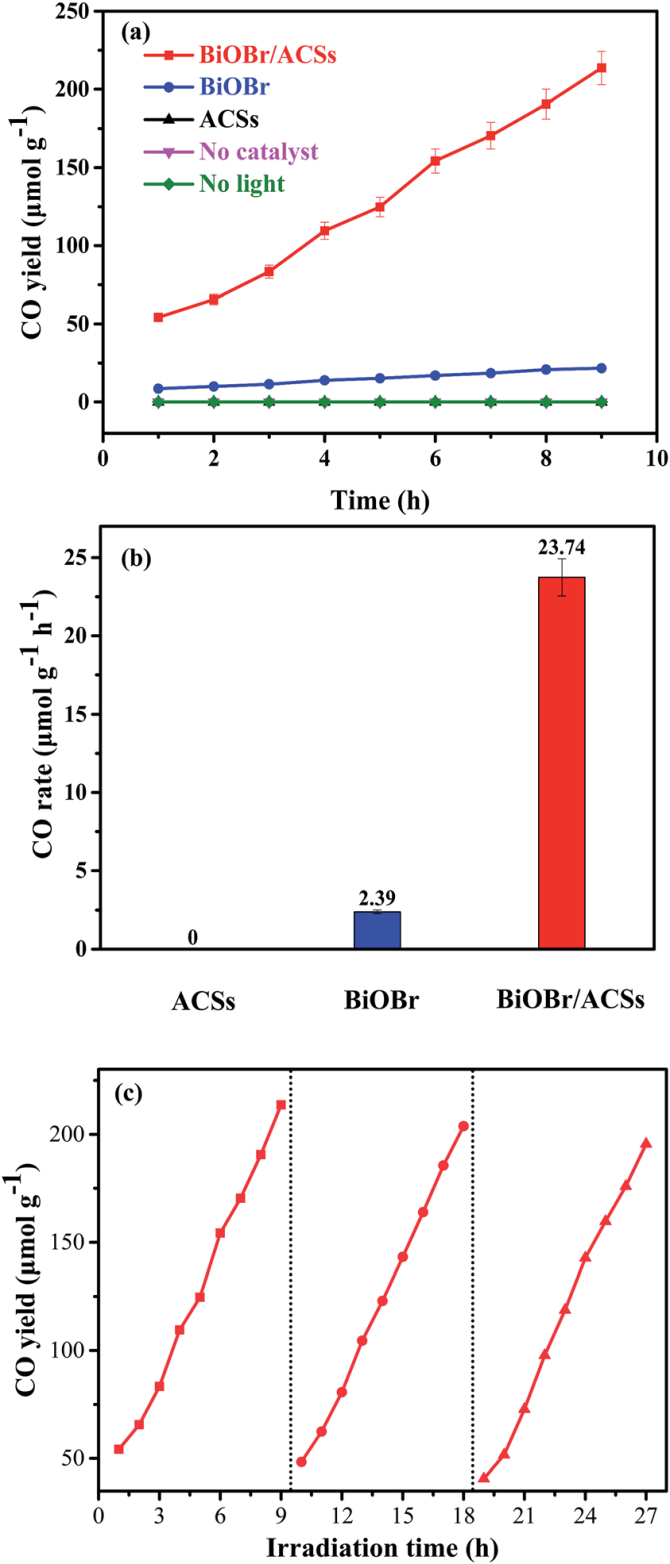
Photocatalytic CO_2_ reduction over BiOBr and BiOBr/ACSs samples under UV-vis irradiation: (a) CO yield, (b) CO rate and (c) cyclic experiments.

Fig. 6 shows the plots of a series of experiments for the photocatalytic CO_2_ reduction over ACSs, BiOBr/ACSs and BiOBr samples under simulated sunlight irradiation. It can be found that CO_2_ cannot be reduced to CO in the absence of the BiOBr photocatalyst or in the dark during these controlled experiments. Only a small amount of CO gas was detected in the reaction system when BiOBr was used as a single photocatalyst under simulated sunlight irradiation (Fig. 6a). Surprisingly, the BiOBr/ACSs sample exhibits significant photocatalytic CO_2_ reduction activity and has fast-raised CO gas production efficiency with an increase in the irradiation time, with the CO yield of 213.67 μmol g^−1^ after 9 h, about 9.9 times that of pure BiOBr (21.51 μmol g^−1^). For the BiOBr/ACSs photocatalyst, the enhanced performance of photocatalytic CO_2_ reduction should mainly be related to the lower electron–hole charge recombination rate (Fig. 4b), higher micropore surface area (792.56 m^2^ g^−1^) and larger micropore volume that can provide more physical activation sites for CO_2_ adsorption (Fig. 5).

Furthermore, the relevant cyclic experiments (Fig. 6c) further reveal that the photocatalytic activity of the BiOBr/ACSs sample only decreases about 8.28% after three cycles (9 h per cycle). Thus, our as-prepared BiOBr/ACSs sample should exhibit high activity, stability and reusability for photocatalytic CO_2_ reduction.

### 3.7 Photocatalytic mechanism

**Fig. 7:**
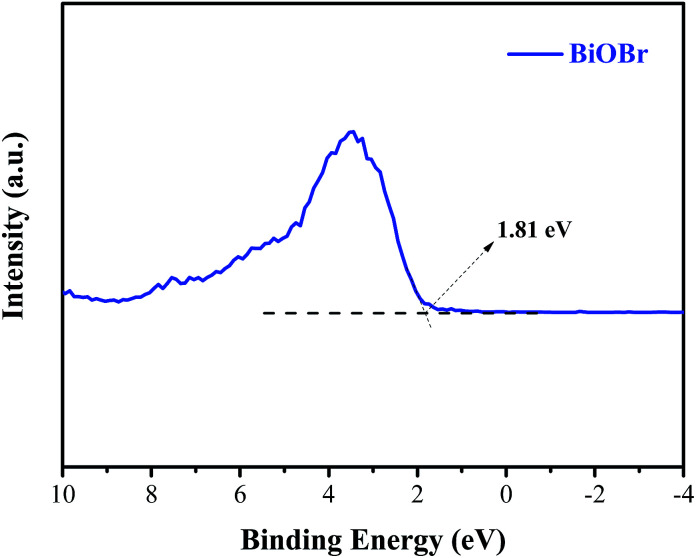
Valence-band XPS spectra of BiOBr.

Generally, the conduction band minimum (CBM) of a semiconductor decided its photocatalytic reduction ability, and a higher CBM will endow the semiconductor with better photocatalytic reduction ability.^20^Fig. 7 shows the valence band (VB) XPS spectra of the as-prepared BiOBr sample. It is found that the VB maximum of BiOBr is 1.81 eV. Combined with the DRS data of *E*_g_ (about 2.36 eV) for BiOBr, the calculated CBM should be −0.55 eV (obtained using the equation *E*_CB_ = *E*_VB_ − *E*_g_),^1^ higher than the *E*_0_(CO_2_/CO) value of −0.53 eV. This is the main reason why a certain photocatalytic CO_2_ reduction activity (2.39 μmol g^−1^ h^−1^) of pure BiOBr exists.

**Scheme 2:**
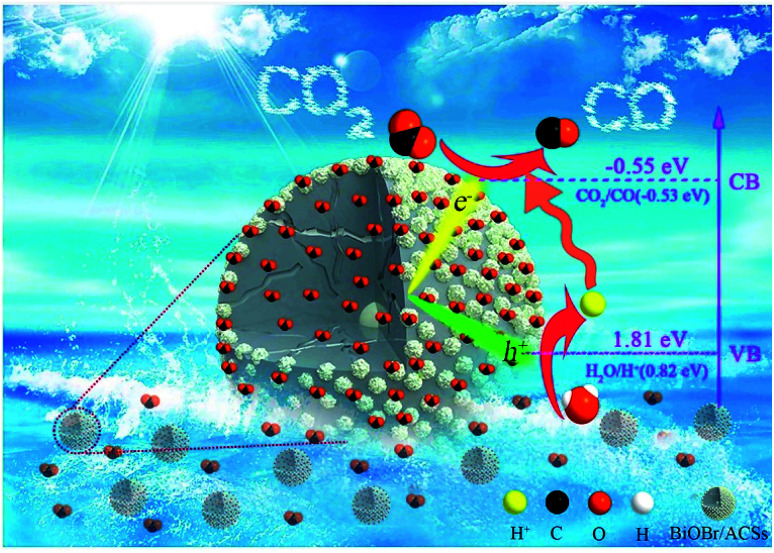
Possible mechanism of the photocatalytic CO_2_ reduction to CO over BiOBr/ACSs.

Based on the abovementioned experimental results and theoretical analyses, a possible mechanism for the enhanced photocatalytic activity of BiOBr/ACSs has been proposed in Scheme 2. As is known, the photocatalytic conversion of CO_2_ to CO should belong to a two-electron reduction reaction process at the gas–solid interface,^4,46^ where CO_2_ adsorption on the catalyst surface and the efficient separation of photoinduced e^−^–h^+^ pairs are two rather important factors affecting the CO_2_ reduction reaction. At first, BiOBr/ACSs can adsorb large amounts of CO_2_ when the system is filled with CO_2_ gas; this is mainly attributed to the contribution of a sufficient reaction zone from ACSs with larger specific surface area and higher pore volume than that of pure BiOBr (Table 1); consequently, a higher number of CO_2_ physical adsorption sites are achieved on the catalyst surface *via* the assistance of ACSs. Then, under simulated sunlight irradiation, BiOBr/ACSs have a more efficient e^−^–h^+^ pair separation than BiOBr (as seen in Fig. 4b); this should be related to the intrinsic electron transport capacity of ACSs,^47,48^ bringing about more efficient electron transfer to avoid the fast recombination of photoinduced electron–hole pairs. Finally, the valence band (VB) electrons (e^−^) of BiOBr are excited to its CBM, and CO_2_ activation is achieved; moreover, the holes (h^+^) left in VBM react with H_2_O to provide H^+^ (h^+^ + H_2_O → H^+^ + 1/2O_2_) for the reduction of CO_2_ to CO (2H^+^ + CO_2_ + 2e^−^ → 2CO + H_2_O). Therefore, our findings on the enhanced photocatalytic CO_2_ reduction activity of BiOBr assisted by ACSs should provide new ideas and an interesting guidance for the construction of high-efficiency photocatalyst systems in the field of CO_2_ reduction to useful high-energy fuels.

## 4. Conclusions

In summary, phenolic resin spheres were prepared by suspension polymerization, and the ACSs were obtained *via* carbonization and steam activation; moreover, the BiOBr/ACSs photocatalyst was built using ACSs as a carrier. The photocatalytic results showed that BiOBr/ACSs had highest photocatalytic activity, with a yield of 213.67 μmol g^−1^ after 9 hours, about 9.9 times that of BiOBr (21.51 μmol g^−1^), and a corresponding CO production rate of approximately 23.74 μmol g^−1^ h^−1^ and 2.39 μmol g^−1^ h^−1^. Through the characterization of the prepared samples, we found that the following excellent characteristics of ACSs improved the ability of BiOBr to reduce CO_2_: (1) ACSs had no effect on the phase structure of BiOBr; (2) the rich pore structure (>83% micropore ratio) and high specific surface area (936.42 m^2^ g^−1^) of ACSs could provide more CO_2_ adsorption sites on the catalyst surface and adsorb more CO_2_ molecules (10.03 mg g^−1^); this allowed BiOBr to be under a CO_2_-enriched atmosphere; (3) the addition of ACSs could reduce the recombination rate of photo-generated electron–hole pairs, improving the effective electron mobility of BiOBr and thus enhancing the photocatalytic activity. Our findings should provide a new insight and method for the design and construction of highly active Bi-based photocatalytic materials assisted by ACSs.

## Conflicts of interest

There are no conflicts to declare.

## Supplementary Material

RA-009-C9RA01329F-s001
