# Supplementary material for: Enhanced photocatalytic reduction of CO2 to CO over BiOBr assisted by phenolic resin-based activated carbon spheres
Source: RSC Adv. 2019 May 8;9(25):14391–9. doi: 10.1039/c9ra01329f (PMC9064128; doi:10.1039/c9ra01329f)

Electronic Supplementary Material (ESI) for RSC Advances.
This journal is © The Royal Society of Chemistry 2019

Electronic Supplementary Information

Enhanced photocatalytic CO_2 reduction to CO over BiOBr assisted by phenolic resin-based activated carbon spheres

Kangli Liu,[a] Xiaochao Zhang,*[a] Changming Zhang,[a] Guangmin Ren,[a] Zhanfeng Zheng,[b] Zhiping Lv,*[a] and Caimei Fan,*[a]

[a]College of Chemistry and Chemical Engineering, Taiyuan University of Technology, Taiyuan 030024, PR China

[b]State Key Laboratory of Coal Conversion, Institute of Coal Chemistry, Chinese Academy of Sciences

Corresponding author:

Xiaochao Zhang, **Zhiping Lv**, **Caimei Fan**, College of Chemistry and Chemical Engineering, Taiyuan University of Technology, Taiyuan 030024, PR China. **Tel.**: +86-15503477962; **Fax**: +86-351-6018554. **E-mail**: zhangxiaochao@tyut.edu.cn; lzping8138@163.com; fancaimei@tyut.edu.cn.

Fig. S1. Photographs of as-prepared (a) ACSs and (b) BiOBr/ACSs samples, (c) low-magnification SEM images of BiOBr/ACSs sample, (d) the high-magnification and (e) the partial enlarged SEM images of selected area for BiOBr/ACSs sample.

Fig. S2. The EDX spectra of each atom for as-prepared BiOBr/ACSs sample.

Fig. S3. Electrochemical impedance spectroscopy of BiOBr and BiOBr/ACSs samples.

Fig. S1.

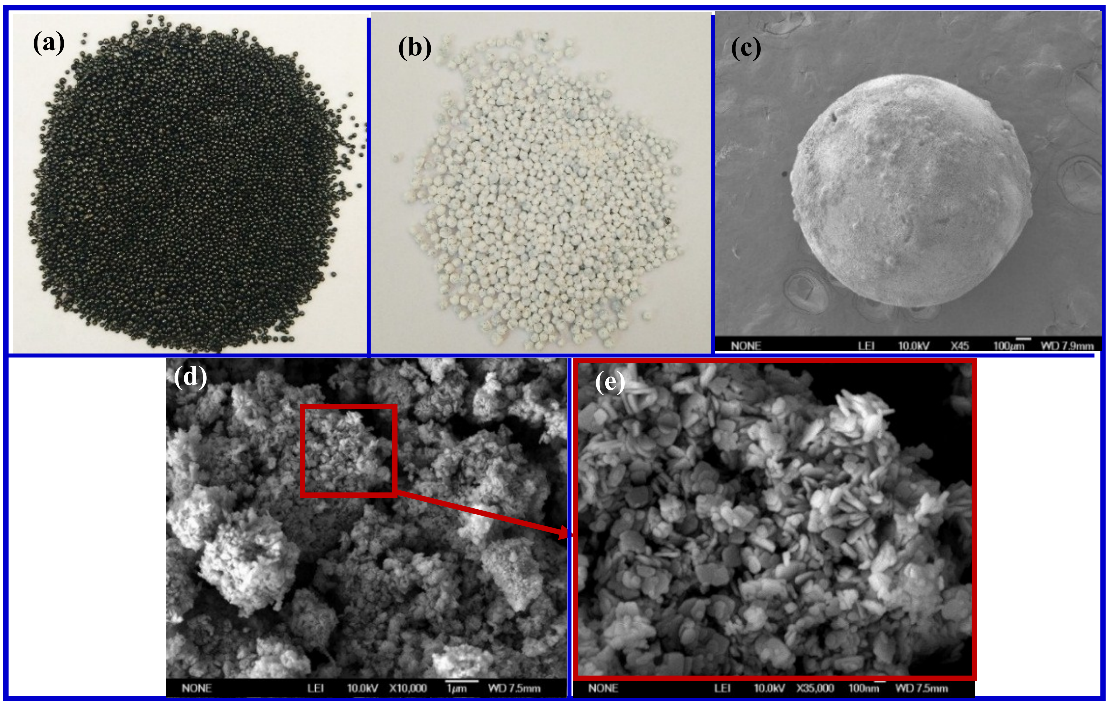
(a)
(b)
(c)
NONE LEI 10.0kV X45 100µm WD 7.9mm
(d)
NONE LEI 10.0kV X10,000 1µm WD 7.5mm
(e)
NONE LEI 10.0kV X35,000 100nm WD 7.5mm

Fig. S2.

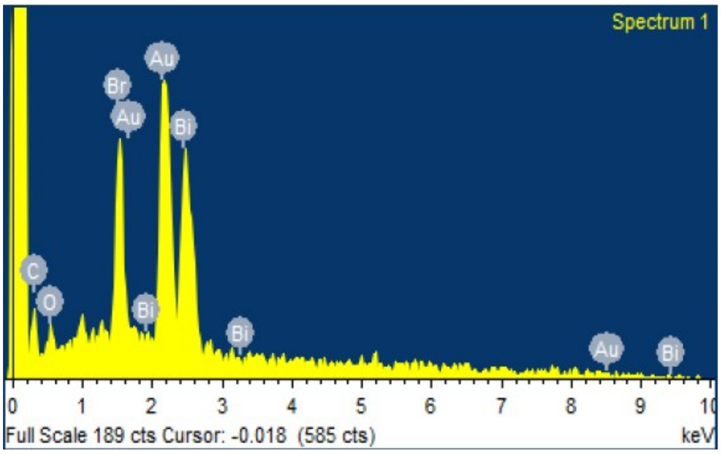

Element	Weight%	Atomic%
C K	6.56	43.13
O K	2.70	13.34
Br L	13.56	13.40
Au M	41.49	16.64
Bi M	35.70	13.49
Totals	100.00	

Electrochemical impedance spectroscopy tests was carried out in a trielectrode quartz chamber system containing 1 mol/L Na_2SO_4 electrolyte detected by electrochemistry workstation (Shanghai Chen Hua CHI600E) under 0.5 V bias. The platinum electrode, Ag/AgCl were used as auxiliary electrode and reference electrode. The frequency range of the electrochemical impedance spectroscopy test is 0.1 Hz-100 kHz.

Fig. S3.

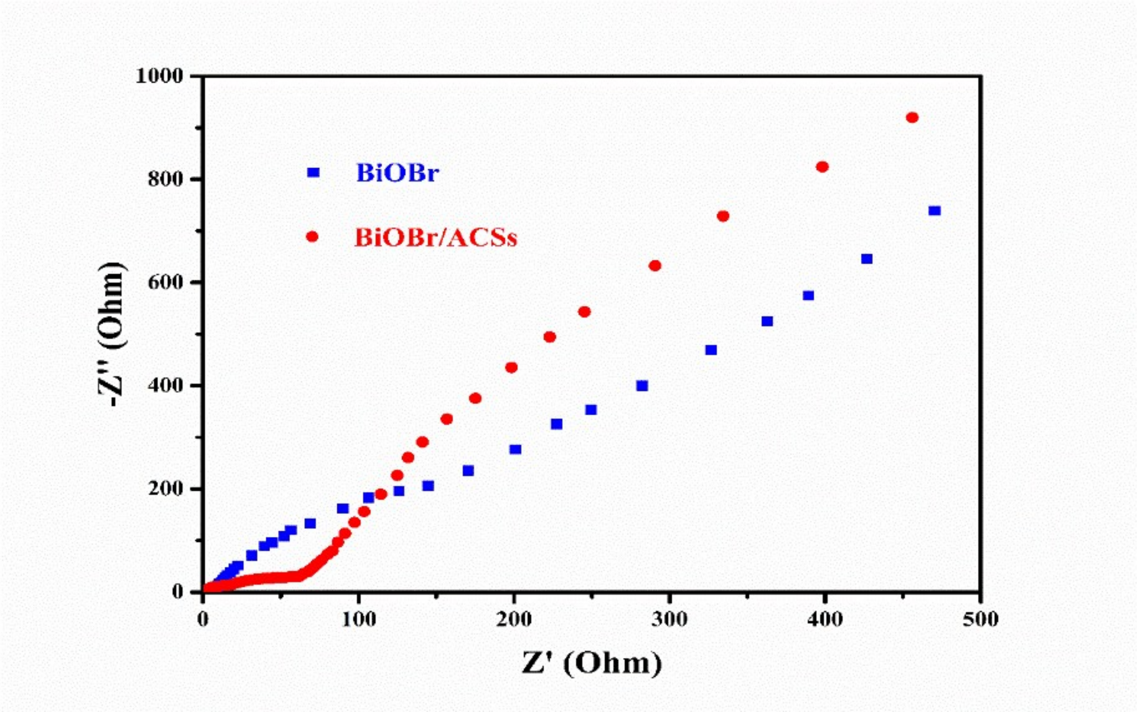

Supplement: RA-009-C9RA01329F-s001 [file RA-009-C9RA01329F-s001.pdf]
